# RNA-Seq transcriptome data of undifferentiated and differentiated gonads of Siberian sturgeon

**DOI:** 10.1016/j.dib.2020.105741

**Published:** 2020-05-22

**Authors:** Christophe Klopp, André Lasalle, Santiago Di Landro, Denise Vizziano-Cantonnet

**Affiliations:** a INRA, SIGENAE, Genotoul Bioinfo, MIAT UR875, Chemin de Borde-Rouge - Auzeville BP 52627, 31326 CASTANET-TOLOSAN CEDEX, France; b Laboratorio de Fisiología de la Reproducción y Ecología de Peces, Instituto de Biología, Facultad de Ciencias, Universidad de la República Oriental del Uruguay, Iguá 4225, Montevideo 11400, Uruguay

**Keywords:** RNA sequencing, gonads, *Acipenser baerii*, sex differentiation, gametogenesis

## Abstract

RNA-Seq transcriptome data from twenty Siberian sturgeon gonads at different developmental stages is described: ten undifferentiated gonads, six gonads of immature males and four gonads from immature females. Siberian sturgeon, *Acipenser baerii*, is long-lived, late-maturing fish farmed in 50 countries but its production remains on a craftsman scale when compared to industrial species. Sturgeon genetic and physiological studies are less developed than for industrial fish. The data presented hereafter enables fundamental studies on the regulatory mechanisms of sturgeon gonad development, which can further be applied both in aquaculture and in fundamental research.

**Specifications Table**

**Table:** 

Subject	Biology
Specific subject area	Transcriptomics, sex determination and differentiation
Type of data	Raw RNA-Seq data and assembled reference transcriptome assembly
How data were acquired	Illumina HiSeq 2500
Data format	Raw data and assembly information
Parameters for data collection	Gonads from10 sex undifferentiated fish of 3 to 6 months of age
	Gonads from 4 immatures males (only spermatogonias) before first maturity and from 6 immatures females (with oogonias and pre-vitellogenic oocytes)
Description of data collection	Gonads were collected from Siberian sturgeons farmed at Estuario del Plata (San Gregorio de Polanco, Uruguay) under natural conditions. The gonad stage was identified by histology for fish ≥6 months of age.
Data source location	Laboratorio de Fisiología de la Reproducción y Ecología de Peces, Facultad de Ciencias, Universidad de la República Oriental del Uruguay.
	Country: Uruguay
	Site of collection: Estuario del Plata sturgeon farm, San Gregorio de Polanco, Tacuarembó, Uruguay
Data accessibility	Raw data of RNA Seq analysis are available on Sequence Read Archive (SRA) database and connected with BioProject PRJNA589957 https://www.ncbi.nlm.nih.gov/bioproject/PRJNA589957 Reference transcriptome assembly is available at the NCBI in TSA https://www.ncbi.nlm.nih.gov/nuccore/GICD00000000

**Value of data**

This data set will benefit the community of scientists working on sex determination/sex differentiation mechanisms and gametogenesis as well as fish evolution.

This data set includes RNA-Seq of gonads at several stages before and after sex differentiation enabling to better understand the control of sex differentiation.

This data will facilitate the development of sex control strategies to increase aquaculture production of Siberian sturgeon.

## 1 Data

Gonadal development, from sex differentiation to the end of gametogenesis and gamete production is a key process for species perpetuation. Understanding the different regulation steps of gonad development is a key point in reproduction studies. These studies may have an impact at the applied level in the control of sex and production of gametes in aquaculture; and they also can be useful for the understanding of the general mechanisms of sex differentiation and its evolution. The Siberian sturgeon is a long lived and late maturing fish for which mechanisms of sex differentiation and gametogenesis are not well understood [1]. The unraveling of the molecular control of sex differentiation and early gametogenesis can profit from gonad RNA-seq data sets at different maturation stages. We provide here a high-quality data set including 10 gonads from sex undifferentiated animals and 10 immature gonads at early stages of gametogenesis.

Gonad samples were taken from fish at several stages of sex undifferentiated period (2.5, 3, 5, and 6 months of age), males at immature stage containing only spermatogonia (8, 9, 14 and 17 months of age), and females with oogonias and different stages of oocyte development previous to vitellogenesis (9 and 17 months of age) [1]. The gonads were sequenced individually using Illumina HiSeq 2500, except at 2.5 months for which gonads of 13 fish were collected individually under binocular loupe and pooled before RNA extraction. Raw data correspond to Fastq for RNA-Seq reads and fasta for assembled contigs. The library identification and the corresponding SRA files are given in the Table 1.

**Table 1:** Fish identification (Id), library name, SRA files, SAMN files, sequencer and quality of sequences produced

Fish Id	Tissue samples	Library name	SRA files	SAMN files	Sequencer	Nb of bases (1)	Alignment rate (2)	Q20 ratio (3)
I1	Undifferentiated gonad	186	SRR10466921	SAMN13294989	Illumina HiSeq 2500	2590499400	93,25	96,19
I2	Undifferentiated gonad	414	SRR10466920	SAMN13294990	Illumina HiSeq 2500	3599333200	95,09	90,54
I3	Undifferentiated gonad	417	SRR10466909	SAMN13294991	Illumina HiSeq 2500	2951092800	94,45	90,50
I4	Undifferentiated gonad	640	SRR10466908	SAMN13294992	Illumina HiSeq 2500	3356820000	95,26	90,16
I5	Undifferentiated gonad	649	SRR10466907	SAMN13294993	Illumina HiSeq 2500	3016267800	94,51	89,90
I6	Undifferentiated gonad	961	SRR10466906	SAMN13294994	Illumina HiSeq 2500	8362045600	94,38	90,12
I7	Undifferentiated gonad	302	SRR10466905	SAMN13294995	Illumina HiSeq 2500	1,2866E+10	96,53	96,92
I8	Undifferentiated gonad	306	SRR10466904	SAMN13294996	Illumina HiSeq 2500	2,4061E+10	95,53	96,86
I9	Undifferentiated gonad	313	SRR10466903	SAMN13294997	Illumina HiSeq 2500	1,2309E+10	97,02	96,20
I10	Undifferentiated gonad	214	SRR10466902	SAMN13294998	Illumina HiSeq 2500	1,245E+10	96,73	97,09
M1	Immature testis	152	SRR10466919	SAMN13294999	Illumina HiSeq 2500	1,5511E+10	95,72	96,40
M2	Immature testis	165	SRR10466918	SAMN13295000	Illumina HiSeq 2500	5879588200	94,92	96,54
M3	Immature testis	172	SRR10466917	SAMN13295001	Illumina HiSeq 2500	5963519600	96,24	96,72
M4	Immature testis	175	SRR10466916	SAMN13295002	Illumina HiSeq 2500	1,0986E+10	94,63	96,65
M5	Immature testis	259	SRR10466915	SAMN13295003	Illumina HiSeq 2500	2511126800	95,24	96,85
M6	Immature testis	260	SRR10466914	SAMN13295004	Illumina HiSeq 2500	3239050400	95,37	96,59
F1	Immature ovary	171	SRR10466913	SAMN13295005	Illumina HiSeq 2500	3099329000	94,01	96,31
F2	Immature ovary	173	SRR10466912	SAMN13295006	Illumina HiSeq 2500	3117568600	94,18	96,54
F3	Immature ovary	261	SRR10466911	SAMN13295007	Illumina HiSeq 2500	2092346200	97,92	96,51
F4	Immature ovary	262	SRR10466910	SAMN13295008	Illumina HiSeq 2500	2587806600	97,91	96,64

(1) Number of bases

(2) Alignment rate: is the number of sequences aligned on the de novo transcriptome reference divided by the total number of sequences of the sample expressed in percent

(3) Q20 ratio is the number of raw read base pairs having a quality score equal or over 20 divided by the total number of read base pairs of the sample expressed in percent.

## 2 Experimental design, materials, and methods

### 2.1 Ethics statement

Research procedures involving animal experimentation complied with international principles on the use and care of laboratory animals and Uruguayan regulations on animal welfare (Comisión Honoraria de Experimentación Animal: CHEA). The protocol was approved by the “Comisión de Etica en el Uso de Animales” from the Comisión Honoraria de Experimentación Animal CHEA of Uruguay (Authorization Number 006-11).

### 2.2 Experimental animals and rearing procedures

Siberian sturgeon (*Acipenser baerii*) individuals were obtained from a fish farm (Estuario del Plata, Uruguay). The embryos come from Poland, and were hatched and reared at natural temperature at the sturgeon farm [2]. The fish were sacrificed by spinal transection. Gonad samples for transcriptome studies were used individually for RNA extraction, except for the 2.5-month-old individuals, for which gonads of 13 fish were pooled for RNA extraction due to their minute size. The external gonad characteristics were observed for fish aged 2.5–5 months that were considered as sex undifferentiated following previous studies made in our Laboratory [1, 3]. For 6-month-old fish, gonad staging was performed in fish from the same cohort as the fish used for RNA extraction, as the gonads are too small to at this age to allow for RNA extraction and histological gonad staging in the same fish. In fish >6 months of age, one gonad was frozen in liquid nitrogen and stored at -80°C until RNA extraction, and the contralateral gonad was stored in 10% formaldehyde for histological analysis). Histological data were reported previously [3].

### 2.3 RNA extraction, cDNA library construction, and Illumina sequencing

RNA was extracted using the Illustra RNAspin Mini RNA Isolation Kit (GE Healthcare, Little Chalfont, UK) according to manufacturer instructions, and quality was assessed using an Agilent 2100 Bioanalyzer. The cDNA libraries were developed from the total RNA of the individual samples. The RNA samples conformed to the required purity criteria (A260/A230 and A260/A280 >1.8) and quality levels (RIN >8) (Agilent 2100 Bioanalyzer) for library preparations for sequencing. The cDNA libraries were constructed on a Tecan EVO200 liquid handler using the Illumina TruSeq Stranded mRNA sample prep kit for RNA analysis. Briefly, the mRNA molecules containing poly (A) were purified using magnetic poly (T) beads from each total RNA sample. A fragmentation buffer was added to break the mRNA into short fragments with an average length of 155 base pairs (bp) (120–210 bp). From these fragments, the first strand cDNA was synthesized using random hexamer primer. The second cDNA strand was synthesized. After purification and end repair, these short cDNA were ligated to the sequencing adapters (60 bp on each side) and enriched by polymerase chain reaction (PCR, 12 cycles). Libraries were checked using an Agilent High Sensitivity DNA Kit and quantified with a KAPA Library Quantification Kit to ensure accuracy. RNA-seq experiments were performed on the Illumina HiSeq 2500 platform (high-throughput mode) with a paired-end read length of 2 × 100 bp and an Illumina TruSeq SBS kit, v3.

### 2.4 Transcriptome assembly

The reads were assembled using the methods presented in [1] and released at in NCBI in TSA (Transcriptome Shotgun Assembly): https://www.ncbi.nlm.nih.gov/nuccore/GICD00000000. The quality assessment results of the assembled transcriptome were presented in [3] and resumed in Table 1.

## Author contributions

C.K. and D.V.C. conceived and designed the experiments; D.V.C directed the research project, acquired the funding and administrate the projects; A.L. and SDL performed the experiments; C.K. and D.V.C. analyzed the data and organized the datasets; the manuscript was written by C.K. and D.V.C.

## Funding

This work was supported by the Comisión Sectorial de Investigación Científica (CSIC), Universidad de la República Oriental del Uruguay, Grant C225-348-Uruguay; the Agencia Nacional de Investigación e Innovación-Dirección Nacional de Recursos Acuáticos ANII-DINARA – FPA 9975-Uruguay; and Agencia Nacional de Investigación e Innovación - FMV_1_2017_1_135908.

## Declaration of Competing Interest

The authors declare that they have no known competing financial interests or personal relationships that could have appeared to influence the work reported in this paper

## Appendix Supplementary materials

Image, application 1
